# Medicinal Plants: A Promising Therapeutic Approach for Addressing Antimicrobial Resistance

**DOI:** 10.3390/ijms27114804

**Published:** 2026-05-26

**Authors:** Huanxin Zhou, Jinkang Du, Meiyan Jia, Yine Li, Kaiyun Zheng

**Affiliations:** College of Chemical Engineering and Technology, Taiyuan University of Science and Technology, No. 66 Waliu Road, Taiyuan 030024, China; llfsdjk@163.com (J.D.); jiameiyan2026@163.com (M.J.); 18235286802@163.com (Y.L.); ky110551@163.com (K.Z.)

**Keywords:** antibiotic resistance, phytochemicals, mechanisms of antimicrobial resistance, medicinal plants

## Abstract

Antimicrobial resistance (AMR) is a critical global public health crisis that is being exacerbated by widespread misuse of antibiotics and rapid bacterial adaptation. The progressive decrease in antibiotic efficacy is also compounded by a stagnating drug-discovery pipeline and underscores the urgent need for innovative and sustainable antimicrobial strategies. This review systematically delineates the core molecular mechanisms driving bacterial resistance, including enzymatic drug inactivation, target modification, reduced membrane permeability, and multidrug efflux pump overexpression. Furthermore, the potential of (flavonoids, alkaloids, and phenolics) as structurally diverse plant-derived compounds with multi-target activity is comprehensively assessed. The features of multi-target activity make them promising dual-function agents that may be capable of both direct antimicrobial action and resistance modulation. These natural products have distinct mechanisms from conventional antibiotics, low propensity for resistance, and versatile bioactivity as biofilm disruptors, enzyme inhibitors, and efflux pump blockers. Numerous phytochemicals exhibit potent synergistic effects with available antibiotics by effectively resensitizing resistant pathogens and extending the clinical utility of current antimicrobials. By integrating mechanistic understanding with translational potential, this review discusses phytochemicals as a sustainable resource for developing next-generation antimicrobial strategies as a complementary approach to revitalize therapeutic pipelines and combat multidrug-resistant infections.

## 1. Introduction

The discovery of broad-spectrum antibiotics in the early 20th century marked a transformative advance in modern medicine that has saved countless lives through direct antimicrobial action and reduced risk of secondary infections. However, pervasive overuse and misuse of antibiotics have led to a global crisis of antimicrobial resistance (AMR), which is being exacerbated by environmental antibiotic dissemination and is undermining the foundations of medical practice [1,2,3]. The rising incidence of emerging and re-emerging infectious diseases is increasing the burden of bacterial resistance [4]. Under sustained antimicrobial selection pressure, pathogens have evolved diverse resistance mechanisms that have led to the proliferation of multidrug-resistant (MDR) bacteria, which are a critical concern for global public health [5]. Particular attention has been given to clinically significant pathogens called the “ESKAPE” group: *Enterococcus faecium*, *Staphylococcus aureus*, *Klebsiella pneumoniae*, *Acinetobacter baumannii*, *Pseudomonas aeruginosa*, and *Enterobacter* spp. This attention is due to the group’s propensity to cause severe infections and escalating resistance to first-line antibiotics [6].

The high prevalence of MDR strains in clinical settings complicates therapeutic management and worsens patient outcomes. Bacterial resistance typically occurs through two principal pathways: intrinsic (constitutive) and acquired resistance [7]. Most bacterial species harbor some intrinsic resistance determinants, but acquired resistance that has developed in initially susceptible strains is the primary driver of clinical resistance [8]. This phenomenon is especially pronounced in immunocompromised populations, such as patients with diabetes, organ transplant recipients, and individuals living with HIV, whose heightened susceptibility to infection accelerates the development of resistance [9]. The extensive use of antibiotics in animal husbandry has also facilitated the environmental spread of resistance genes and has increased the rate of bacterial adaptation [10].

Bacterial adaptation to antibiotics is enabled by high genetic plasticity [11]. Pathogens can acquire resistance to nearly all classes of clinically used antibiotics through adaptive mutations, horizontal gene transfer, or transcriptional reprogramming [12]. Key molecular mechanisms include enzymatic drug inactivation, active efflux, target modification, and impaired drug uptake [13]. Once established, these resistance traits can propagate vertically to progeny or horizontally across species boundaries, which greatly complicates therapeutic efforts [14,15].

The escalating AMR crisis has occurred in parallel with a decline in antibiotic research and development. There are currently three interrelated challenges: (1) Traditional discovery approaches based on environmental screening have yielded decreasing returns, which has limited the pipeline of novel antimicrobial chemotypes [16]. (2) Inadequate economic incentives have driven most pharmaceutical companies to abandon the research and development of antibiotics resulting in a severe shortage of new lead compounds [17]. (3) Research funding is increasingly allocated to therapeutic areas with higher financial returns. By the end of 2020, only 43 antibiotics were in phase I–III clinical trials, compared with over 1300 anticancer agents [18]. This inadequate innovation has widened the therapeutic gap for drug-resistant infections and highlights the need for alternative antimicrobial sources.

Medicinal plants are a promising reservoir of novel antibacterial agents. Long before the era of antibiotics, plant-based preparations were widely employed to treat infectious diseases. Plants produce a diverse array of secondary metabolites as chemical defenses, and many of them have potent antibacterial activity and remain integral to traditional medicine [19]. These phytochemicals can act as standalone antimicrobial agents or as potentiators that restore the activity of conventional antibiotics against MDR pathogens [20]. Their structural and functional diversity encompasses a broad spectrum of antimicrobial mechanisms that could provide valuable templates for next-generation drug discovery and therapeutic strategies [21]. In general, the antibacterial effects of phytochemicals involve direct bactericidal action, inhibition of essential bacterial targets, or synergy with antibiotics to counteract resistance mechanisms [22].

The present review was designed to systematically delineate the principal molecular mechanisms that drive bacterial resistance to current antibiotics with a focus on enzymatic drug inactivation, target modification, reduced permeability, and multidrug efflux. This study critically evaluates the antibacterial and resistance-modulating activities of phytochemicals and their functions as biofilm disruptors, enzyme inhibitors, and efflux pump blockers. The review also assesses the enhanced therapeutic potential of phytochemicals both individually and in combination with conventional antibiotics against MDR bacterial strains. By integrating mechanistic insights with translational perspectives, this work highlights the role of plant-derived compounds as a viable source of resistance-breaking strategies. This sustainable and complementary approach could reinvigorate the antimicrobial arsenal and address the growing challenge of AMR.

## 2. Mechanisms of Antibacterial Action and Bacterial Drug Resistance

### 2.1. Efflux Pumps

Bacterial multidrug efflux pumps (EPs) are integral membrane transporters that constitute a first-line defense mechanism by actively extruding a wide spectrum of antimicrobial agents from the cell, thereby directly reducing intracellular drug concentration and conferring resistance [23]. The role of EPs extends beyond mere drug resistance, and they are now recognized as integral components of bacterial physiology, particularly in MDR strains, where they significantly influence adaptive fitness and virulence [24].

**Figure 1:**
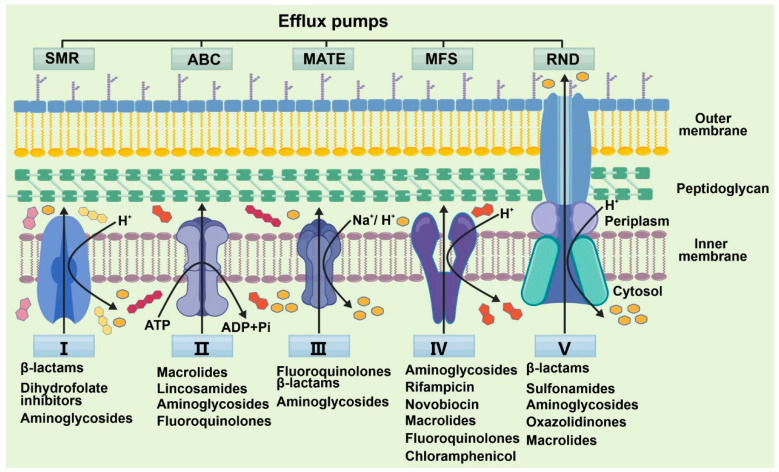
Primary families of bacterial efflux pump classes: (I) the small multidrug resistance (SMR) family; (II) the ATP-binding cassette (ABC) superfamily; (III) the multidrug and toxic compound extrusion (MATE) family; (IV) the major facilitator superfamily (MFS); and (V) the resistance-nodulation-division (RND) family. This diagram was created using BioRender.com.

Based on their energy-coupling mechanisms, bacterial efflux pumps are broadly classified using two major categories: (1) Secondary active transporters harness ion gradients (proton or sodium motive force) and encompass the multidrug and toxic compound extrusion (MATE), major facilitator superfamily (MFS), resistance–nodulation–division (RND), and small multidrug resistance (SMR) families. (2) Primary active transporters of the ATP-binding cassette (ABC) superfamily utilize ATP hydrolysis to drive substrate extrusion [24] (Figure 1). MFS, ABC, SMR, and MATE families are distributed across both Gram-positive and Gram-negative bacteria, but the RND superfamily comprises exclusively Gram-negative organisms [25]. Efflux-mediated antibiotic resistance is widespread among diverse bacterial pathogens.

#### 2.1.1. The SMR Family

SMR transporters are the smallest known efflux systems, typically comprising about 100–140 amino acids arranged into four transmembrane helices [26,27]. Unlike many broader-specificity multidrug transporters, SMR pumps have a relatively narrow substrate profile that is primarily limited to lipophilic compounds such as certain β-lactams, dihydrofolate reductase inhibitors, and aminoglycoside antibiotics [28]. Clinically, SMR pumps contribute to biocide and disinfectant resistance in several pathogens. For instance, they confer resistance to ethidium bromide, acriflavine, benzalkonium, and amikacin in *Acinetobacter baumannii* [29] and enhance tolerance to benzalkonium chloride and chlorhexidine in *Klebsiella pneumoniae* [30]. They also facilitate the extrusion of polycyclic aromatic compounds in *Escherichia coli* and *Pseudomonas aeruginosa* [31].

#### 2.1.2. ABC Transporter Family

ABC transporters are a ubiquitous and functionally diverse superfamily with roles extending beyond drug extrusion to signal transduction, protein secretion, nutrient acquisition, sporulation, and virulence [32]. A defining structural feature is the presence of two conserved nucleotide-binding domains (NBDs) and two transmembrane domains (TMDs), which form the core translocation machinery [33]. In bacteria, these domains may exist as discrete subunits or as fused polypeptides that assemble into functional homo- or heterodimeric complexes [34],with substrate translocation powered by ATP hydrolysis.

Structural and organizational variations are evident between Gram-positive and Gram-negative organisms [35]. In Gram-positive bacteria, representative pumps such as EfrAB, LmrA, Msr, and PatA/B often function as single-polypeptide or dimeric systems [36]. For instance, the heterodimeric pump EfrAB confers resistance to aminoglycosides and phenols [37], while LmrA from *Lactococcus lactis* is a homodimeric transporter (with one NBD per monomer) that exports macrolide and lincosamide antibiotics [38,39]. In *Streptococcus pneumoniae*, ABC transporters contribute to fluoroquinolone resistance by extruding hydrophilic agents such as ciprofloxacin and norfloxacin [40].

#### 2.1.3. The MATE Family

MATE transporters are defined by a conserved architecture of 12 transmembrane segments and typically feature extended N- and C-terminal regions and pseudo-twofold symmetry [41]. These secondary transporters utilize proton or sodium ion gradients to drive substrate efflux and undergo cyclic conformational transitions between outward- and inward-facing states [42,43]. Bacterial MATE proteins preferentially export cationic xenobiotics and toxic compounds, with notable specificity toward β-lactams, fluoroquinolones, and aminoglycoside antibiotics [44,45,46]. Fluoroquinolones in particular are recognized as universal substrates across nearly all members of the MATE family [47].

#### 2.1.4. The MFS Family

The MFS is one of the largest groups of secondary transporters. They typically function as single-component systems in most bacteria, but MFS-type multidrug efflux pumps in Gram-negative organisms often assemble into tripartite complexes that span the entire cell envelope [48,49]. In such assemblies, the MFS transporter associates with a periplasmic adaptor protein and an outer-membrane channel and forms a contiguous conduit for direct drug extrusion [50]. In such assemblies, the MFS transporter associates with a periplasmic adaptor protein and an outer-membrane channel and forms a contiguous conduit for direct drug extrusion [47]. Beyond their role in AMR, MFS transporters participate in diverse physiological processes, including quorum sensing, osmotic stress adaptation, solute transport, and biofilm formation, thereby increasing bacterial fitness and virulence [51,52]. These pumps are energized by the proton motive force and export a broad spectrum of cationic agents, including macrolides, rifampicin, aminoglycosides, chloramphenicol, fluoroquinolones, novobiocin, and quinolones [53].

#### 2.1.5. The RND Superfamily

The RND superfamily is evolutionarily conserved and found in both prokaryotes and eukaryotes. It is characterized by a tripartite architecture comprising an inner-membrane transporter, a periplasmic membrane fusion protein, and an outer-membrane channel [54]. Although widely distributed, RND-type efflux systems are most prominently associated with Gram-negative bacteria, where they actively export a diverse array of antimicrobial agents and toxic compounds [55]. Clinically, RND pumps are major contributors to multidrug resistance in pathogens such as *Escherichia coli*, *Acinetobacter baumannii*, and *Pseudomonas aeruginosa* and are also prevalent in *Campylobacter jejuni*, *Neisseria gonorrhoeae*, *Klebsiella pneumoniae*, *Salmonella enterica*, *Stenotrophomonas maltophilia*, and *Pseudomonas putida* [56,57].

These systems form a continuous transmembrane channel that captures substrates from the inner membrane and directly translocates them to the extracellular milieu through proton antiport [58]. RND pumps exhibit broad substrate specificity and recognize antibiotics with divergent chemical structures. Their overexpression is a key determinant of multidrug resistance in clinical isolates [24,59]. The archetypal RND pump AcrB associates with the outer-membrane protein TolC and the periplasmic adaptor AcrA to form a functional tripartite efflux complex [60,61]. Such systems confer intrinsic resistance to numerous lipophilic antibiotics, including β-lactams, sulfonamides, macrolides, aminoglycosides, and oxazolidinones in wild-type Gram-negative strains [62].

EP expression is tightly regulated, primarily by locally encoded repressors, and integrated into broader transcriptional networks that respond to environmental cues via local and global regulators. This dynamic regulation enables bacteria to adjust pump expression under stress. In clinical isolates, mutations in these regulatory genes commonly lead to constitutive overexpression and enhanced resistance, positioning EPs as active components of bacterial survival rather than passive resistance determinants. Deciphering these circuits may therefore reveal novel therapeutic targets [63,64,65,66,67].

The structural and functional complexity of EPs, particularly the promiscuous substrate binding pockets of pumps like AcrB and MexB, makes them high-value yet challenging targets. They are uniquely susceptible to interference by small, hydrophobic plant-derived molecules. Phytochemicals such as flavonoids and alkaloids can act as competitive inhibitors or allosteric modulators, directly antagonizing efflux and thereby reversing a major mechanism of multidrug resistance. This pharmacological strategy forms a cornerstone of phytochemical-based adjuvant therapy, offering a promising yet underexploited avenue for combating MDR infections.

### 2.2. Bacterial Protein Biosynthesis as a Major Antibiotic Target

**Figure 2:**
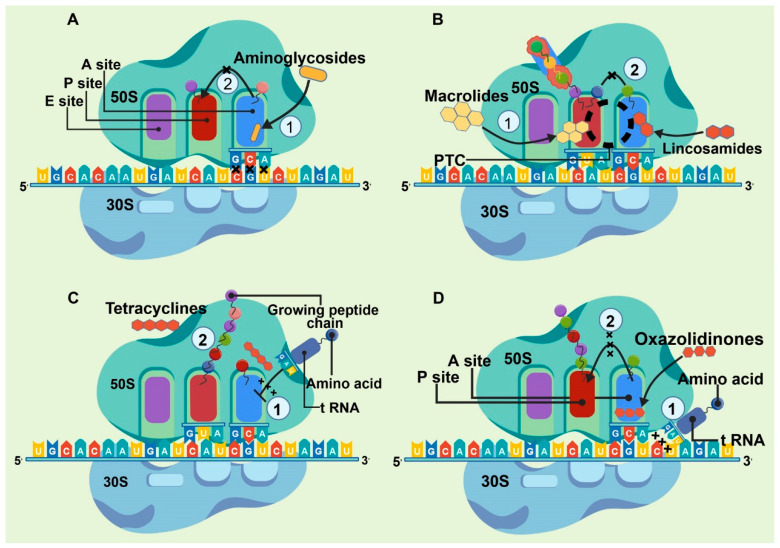
Modes of action of different classes of antibiotics: (**A**) Aminoglycosides have two primary mechanisms: ① disrupting codon recognition while inhibiting ribosomal translocation and ② blocking the translocation of peptidyl-tRNA from the A-site to the P-site of the ribosome. (**B**) Macrolides and lincosamides share a similar mechanism of action: ① binding to the PTC and obstructing the ribosomal exit tunnel and ② impeding the proper positioning of A-site tRNA, leading to inhibition of protein biosynthesis. (**C**) Tetracyclines act through two distinct pathways: ① binding to specific molecular targets on the 30S ribosomal subunit to suppress the translation process and ② competing with tRNA for binding to the ribosomal P-site and suppressing polypeptide chain elongation. (**D**) Oxazolidinones inhibit bacterial protein synthesis by: ① binding to the A-site of the 50S ribosomal subunit and interfering with translation initiation, as well as ② preventing tRNA translocation from the A-site to the P-site and blocking the elongation phase. This figure was created with BioRender.com.

As the primary clinically validated target for therapeutic antibiotics, the bacterial ribosome harbors three critical functional sites that are exploited by distinct drug classes [68,69]: (1) The A-site (aminoacyl-tRNA site) on the 30S subunit, disruption of which impairs codon recognition and tRNA translocation; (2) The peptidyl transferase center (PTC) on the 50S subunit, inhibition of which suppresses peptide bond formation; and (3) The nascent peptide exit tunnel (NPET) on the 50S subunit, blockade of which impedes chain elongation and release. The inhibition of protein synthesis through these sites is a cornerstone of antibacterial therapy. The mechanisms that target these vulnerabilities are discussed in the context of major antibiotic classes: aminoglycosides, macrolides, tetracyclines, lincosamides, and oxazolidinones [70] (Figure 2).

(1) Aminoglycosides act by binding to the A-site on the 30S ribosomal subunit and interfering with codon recognition and tRNA translocation [71] (Figure 2A). In physiological conditions, the amino groups of aminoglycosides engage in electrostatic and hydrogen-bonding interactions with 16S rRNA [72]. Specific nucleotides are critical for high-affinity drug binding (notably adenosine 1408 (A1408) and guanine 1491 (G1491) in 16S rRNA). Structural studies indicate that ring I of the aminoglycoside core stacks against G1491 and forms hydrogen bonds with A1408, which stabilizes the decoding center in a conformation that compromises translational fidelity [73]. Point mutations such as A1408G reduce susceptibility to aminoglycosides [74], and in organisms with a single rRNA operon, this single mutation can confer high-level resistance [75]. Beyond inducing miscoding, certain aminoglycosides also inhibit translation by interfering with initiation complex assembly or blocking translocation of the peptidyl-tRNA from the A- to the P-site [4,76,77].
(2) Macrolides have a central macrolactone ring and inhibit protein synthesis by binding to the nascent peptide exit tunnel (NPET) of the 50S ribosomal subunit, which is adjacent to the PTC, the catalytic hub for peptide bond formation [78,79,80,81]. Clinically relevant agents such as erythromycin and azithromycin bind specifically to domain V of the 23S rRNA (notably interacting with nucleotide A2058), which leads to physical occlusion of the tunnel lumen and sterically hinderance of the progression of the nascent polypeptide chain [82]. During elongation, the nascent peptide transits a dynamic rRNA-lined tunnel below the PTC prior to its exit [83,84], and peptide sequences can modulate tunnel gating and induce translational pausing [85]. Macrolide binding exacerbates this effect by inducing sequence-dependent ribosome stalling [86]. Rather than universally stopping all translation, macrolides primarily arrest ribosomes when specific amino acid motifs (e.g., certain charged or bulky residues) attempt to pass the drug-bound constriction, which leads to peptidyl-tRNA drop-off and abortive termination of protein synthesis [87].
(3) Lincosamides, including lincomycin and its semi-synthetic derivative clindamycin, inhibit bacterial protein synthesis by targeting the 50S ribosomal subunit in a manner that functionally overlaps with macrolides, although they are structurally distinct. Structural studies indicate that lincosamides bind at the PTC, where they sterically occlude the A-site and partially overlap with the P-site [88]. Rather than precisely mimicking full tRNA molecules, these antibiotics are proposed to occupy the space that normally accommodates the CCA end of A-site tRNA, which prevents its correct positioning and inhibits peptide bond formation [89]. Clindamycin has higher potency and further suppresses translation by interfering with the initial binding and accommodation of aminoacyl-tRNA in the A-site, which effectively blocks the peptidyl transferase reaction [90,91].
(4) Tetracyclines inhibit bacterial protein synthesis by reversibly binding to the 16S rRNA of the 30S ribosomal subunit and primarily targeting a Mg^2+^-dependent site in helix 34 (h34), which overlaps with the A-site decoding center [92]. This high-affinity interaction sterically blocks the accommodation of aminoacyl-tRNA and arrests translation elongation [93,94] (Figure 2C). A secondary mechanism involves competition with the CCA end of peptidyl-tRNA at the P-site, which perturbs tRNA positioning and further suppresses polypeptide chain elongation [95]. The well-defined structural basis of tetracycline binding is also related to a major resistance mechanism involving ribosome protection proteins (RPPs), such as TetM and TetO [96]. These GTPases bind to the ribosome and catalytically displace tetracycline in a GTP-dependent manner, which leads to high-level resistance through a mechanism that is distinct from efflux or enzymatic inactivation [92,94,97].
(5) Oxazolidinones bind to the A-site of the PTC in the 50S ribosomal subunit, where they directly compete with the aminoacyl moiety of initiator tRNA for binding [98]. This interaction inhibits protein synthesis through two principal pathways: (1) preventing the formation of a functional 70S initiation complex by disrupting the correct positioning of the initiator tRNA [99] and (2) blocking the translocation of peptidyl-tRNA from the A- to the P-site during elongation, which stalls the ribosome [100] (Figure 2D).

In summary, despite its structural conservation and functional centrality, the bacterial ribosome has a limited set of vulnerable nodes: the A-site, PTC, and NPET. The intense evolutionary pressure exerted by antibiotics that target these sites has led to the emergence of sophisticated resistance mechanisms. Thus, the utility of ribosome-targeting drugs is continually challenged. It is important to understand the mechanisms and structures underlying their action to obtain novel compounds that evade common resistance determinants, hybrid antibiotics that engage multiple sites concurrently, and allosteric inhibitors that target unexploited ribosomal interfaces.

The conserved functional sites of the ribosome, while vulnerable to resistance via mutation or methylation, also present opportunities for compounds that bind to novel or adjacent sites. Several phytochemicals, including certain alkaloids and terpenoids, are known to inhibit protein synthesis by interacting with ribosomal subunits at locations distinct from classical antibiotic binding sites. This alternative targeting can bypass common resistance determinants and potentially restore translational inhibition in resistant strains.

### 2.3. Enzymatic Modification and Hydrolysis of Antibiotics

**Figure 3:**
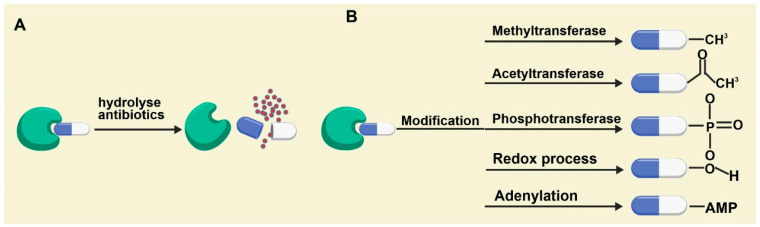
The main enzymatic mechanisms of antibiotic inactivation. (**A**) (1) hydrolysis; (**B**) (1) methylation; (2) acetylation; (3) phosphorylation; (4) based on redox modification; (5) adenylation. The illustration was created with BioRender.com.

Enzymatic inactivation is a versatile primary mechanism of antibiotic resistance that is driven by a diverse and evolutionarily adaptable family of bacterial enzymes [101,102]. These proteins function by directly modifying or hydrolyzing antibiotic molecules and sterically or functionally ablating their interaction with cellular targets. Clinically, the most significant enzymes fall into two broad categories: antibiotic-modifying enzymes and hydrolytic enzymes. Antibiotic-modifying enzymes include aminoglycoside-modifying enzymes (AMEs), aminoglycoside phosphotransferases (APHs), and methyltransferases (MTs), while the most notable hydrolytic enzymes are β-lactamases. These enzymes employ distinct chemical mechanisms, including acetylation, phosphorylation, adenylation, and hydrolysis, which are each tailored to inactivate specific antibiotic scaffolds [103] (Figure 3).

#### 2.3.1. Aminoglycoside-Modifying Enzymes (AMEs)

AMEs confer resistance by covalently modifying hydroxyl or amino groups on the antibiotic, which substantially reduces its binding affinity for the 16S rRNA target in the bacterial 30S ribosomal subunit [104]. AME genes are predominantly located on mobile genetic elements and facilitate rapid horizontal dissemination, although chromosomal integration has been documented in species such as *Providencia stuartii* and *Serratia marcescens* [104]. Aminoglycoside phosphotransferases (APHs) are an AME subclass that utilizes ATP to phosphorylate specific hydroxyl groups. For instance, APH(3′)-IIIa is prevalent in enterococci and staphylococci and confers broad resistance to kanamycin and neomycin [105].

Bifunctional enzymes pose a more formidable clinical challenge. This is exemplified by AAC(6′)-Ie/APH(2″)-Ia in *Staphylococcus aureus*, which combines acetylation and phosphorylation activities that inactivate nearly all clinically relevant aminoglycosides except streptomycin [106]. The plasmid-borne nature of these resistance determinants accelerates their dissemination, promoting the emergence of MDR phenotypes in clinical settings.

#### 2.3.2. Ribosomal Methyltransferases (MTs)

In contrast to antibiotic modification, MTs confer high-level resistance by modifying the drug target. Enzymes such as ArmA and RmtB methylate specific 16S rRNA nucleotides (e.g., G1405, A1408) in the ribosomal decoding site while using S-adenosylmethionine (SAM) as a methyl donor [107,108]. This modification introduces steric hindrance and electrostatic repulsion and effectively blocks aminoglycoside binding while preserving ribosomal function [108]. Although originally a self-defense mechanism in aminoglycoside-producing actinomycetes, genes encoding 16S rRNA MTs (e.g., *armA*, *rmtB*) are now frequently carried on broad-host-range plasmids in high-priority pathogens such as *Klebsiella pneumoniae* and *Acinetobacter baumannii* [107]. A particular concern is that the genes encoding these MTs are often located on broad-host-range plasmids that concurrently carry carbapenemase genes (e.g., *blaNDM-1*). This genetic linkage drives the emergence of pan-resistant isolates that evade all conventional therapeutic regimens.

#### 2.3.3. Hydrolytic Inactivation: β-Lactamases

β-lactam resistance is the most prevalent and clinically significant form of enzymatic resistance and is primarily mediated by β-lactamases. These enzymes inactivate β-lactam antibiotics by hydrolyzing the essential amide bond in the β-lactam ring [109]. The genes encoding β-lactamases are commonly located on mobile genetic elements, including plasmids, transposons, and integrons, which enables efficient horizontal gene transfer among bacterial populations [110]. Based on their molecular structure and catalytic mechanism, β-lactamases are divided into four Ambler classes (A–D). Classes A, C, and D were serine-dependent hydrolases, whereas class B comprises metallo-β-lactamases (MBLs) that require zinc ions for catalytic activity, such as NDM and VIM [109].

Carbapenemases are a particular concern as they are capable of hydrolyzing nearly all β-lactam antibiotics, including carbapenems. When combined with ancillary resistance mechanisms such as outer membrane porin loss or EP upregulation, carbapenemase production can precipitate extensively drug-resistant (XDR) or even pan-drug-resistant (PDR) phenotypes, which critically limits the therapeutic options for life-threatening infections [111]. The evolutionary origins of many of these enzymes can be traced to environmental bacteria (e.g., *Streptomyces* spp.) [112,113], from which their genes have mobilized onto promiscuous genetic platforms, which has enabled their global spread in pathogens.

In summary, the enzymatic arsenal of bacteria demonstrates remarkable mechanistic diversity and evolutionary adaptability and pose a great challenge in antimicrobial therapy. From the covalent modification of antibiotics and their targets to the direct hydrolysis of essential pharmacophores, these resistance determinants are frequently encoded on mobile genetic elements, which facilitate rapid dissemination across species and ecological niches. Addressing this escalating threat necessitates a multifaceted strategy that integrates enhanced global surveillance, stringent antibiotic stewardship, and accelerated development of therapeutic agents, including next-generation β-lactamase inhibitors and compounds that evade enzymatic inactivation.

The catalytic versatility of resistance enzymes like β-lactamases necessitates equally versatile inhibitors. Plant secondary metabolites offer a rich source of such compounds. Many polyphenols and flavonoids exhibit inhibitory activity against both serine and metallo-β-lactamases, often through non-competitive or chelation mechanisms that differ from clinical inhibitors like clavulanate. This chemical diversity is crucial for overcoming evolving enzyme-mediated resistance.

### 2.4. Inhibition of Cell Wall Synthesis and Associated Resistance Mechanisms

A fundamental mechanism of antibiotic resistance is target site modification, achieved primarily through structural alteration or functional bypass of essential proteins. This strategy is often driven by point mutations or the acquisition of alternative enzymes and makes antibiotics ineffective by decreasing drug-binding affinity. This mechanism is employed against various drug classes and is particularly prominent and well characterized in the context of antibiotics that target bacterial cell-wall synthesis, such as β-lactams and glycopeptides [114].

#### 2.4.1. Resistance to β-Lactam Antibiotics

**Figure 4:**
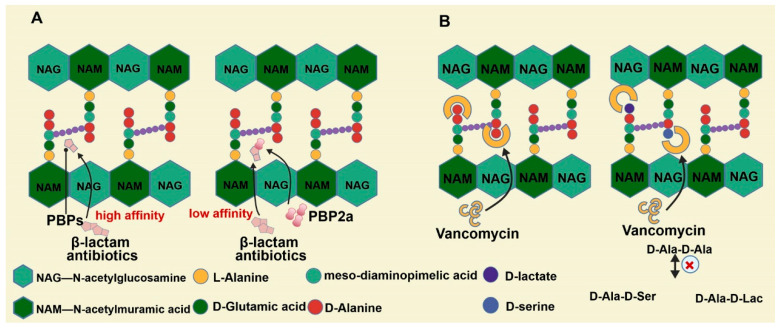
The main enzymatic mechanisms of antibiotic inactivation. (**A**) (1) alteration of penicillin-binding proteins (PBPs); (**B**) (2) modification of peptidoglycan precursor termini.

Resistance to β-lactam antibiotics is commonly mediated by the production of modified penicillin-binding proteins (PBPs) that exhibit low affinity for the drugs. The most clinically significant example is methicillin-resistant *Staphylococcus aureus* (MRSA), which produces PBP2a. This transpeptidase, encoded by the *mecA* or *mecC* gene located on the staphylococcal cassette chromosome mec (SCCmec), a mobile genetic element [115,116,117]. PBP2a retains the transpeptidase activity that is essential for peptidoglycan cross-linking, yet its altered active site greatly reduces β-lactam binding and makes these antibiotics ineffective [118] (Figure 4).

Notably, the more recently identified mecD gene in *Macrococcus caseolyticus* results in broad-spectrum resistance to β-lactams, including cephalosporins [119]. An alternative resistance strategy involves bypassing the classical PBP-dependent pathway altogether. In Escherichia coli, for instance, the bacterium can utilize the L, D-transpeptidase YcbB to perform cell-wall cross-linking independently of the native PBPs and evade β-lactam inhibition [120,121].

#### 2.4.2. Resistance to Glycopeptide Antibiotics

Glycopeptide antibiotics such as vancomycin target a later stage of cell-wall biosynthesis. They act by forming high-affinity complexes with the D-alanyl-D-alanine (D-Ala-D-Ala) termini of lipid-linked peptidoglycan precursors and blocking the trans-glycosylase and transpeptidase activities that are essential for polymer assembly [122]. The primary resistance mechanism involves target modification through the enzymatic substitution of the terminal D-Ala residue in the pentapeptide precursor with either D-serine (D-Ser) or D-lactate (D-Lac). This alteration greatly reduces antibiotic binding as the affinity for the modified target is decreased by approximately seven-fold (D-Ala-D-Ser) to 1000-fold (D-Ala-D-Lac), which effectively abolishes drug activity [123,124] (Figure 4). These modifications are orchestrated by specific resistance gene clusters, particularly the *van* operons, which enable pathogens like vancomycin-resistant enterococci (VRE) and certain MRSA strains to maintain cell-wall synthesis in the presence of the antibiotic.

Target modification, exemplified by PBP2a in MRSA, directly alters the antibiotic’s binding site. Phytochemicals can counteract this resistance not by competing for the same target, but through polypharmacological actions. Certain plant-derived compounds can downregulate the expression of resistance genes (e.g., *mecA*) or inhibit accessory enzymes involved in cell wall biosynthesis, thereby weakening the resistance phenotype at regulatory or metabolic levels and effectively restoring bacterial susceptibility to the primary antibiotic.

### 2.5. Adaptations Limiting Antibiotic Permeability and Access

To limit antibiotic penetration, bacteria have evolved sophisticated structural and physiological adaptations that constitute a major determinant of intrinsic resistance, particularly in Gram-negative pathogens [125]. These adaptations function as a dual-layered defensive strategy, comprising an intrinsic cell-envelope barrier and an inducible, population-level biofilm barrier. Together, they critically limit antimicrobial access to intracellular targets and greatly reduce therapeutic efficacy.

#### 2.5.1. The Cellular Envelope Barrier: Outer Membrane and Porins

The outer membrane (OM) of Gram-negative bacteria is a formidable selective permeability barrier that confers resistance to diverse antimicrobial classes, including many β-lactams, tetracyclines, and hydrophilic fluoroquinolones [126,127]. Its lipopolysaccharide (LPS)-enriched outer leaflet is a particular challenge for the penetration of large or amphipathic molecules. This is exemplified by glycopeptides such as vancomycin (>1400 Da), which exhibit negligible activity against most Gram-negative organisms due to their inability to traverse this barrier [128]. Porins are β-barrel channel proteins critical for OM permeability. They facilitate the passive diffusion of small hydrophilic solutes (<600 Da) and serve as primary entry points for many antibiotics [129]. Alterations in porin expression or structure are a well-established mechanism of acquired resistance [130].

Mutations in porin-encoding genes (e.g., *omp* in *Escherichia coli*) can modify channel properties and reduce the influx of key antibiotics such as cefotaxime, gentamicin, and imipenem [131]. Porin expression is also dynamically regulated. For instance, under osmotic stress, the EnvZ/OmpR two-component system remodels membrane permeability: the sensor kinase EnvZ phosphorylates the response regulator OmpR, which then represses the general porin OmpF while upregulating the narrower channel OmpC, collectively reducing overall antibiotic influx as an adaptive response [132,133].

#### 2.5.2. Biofilms: A Population-Level Barrier

**Figure 5:**
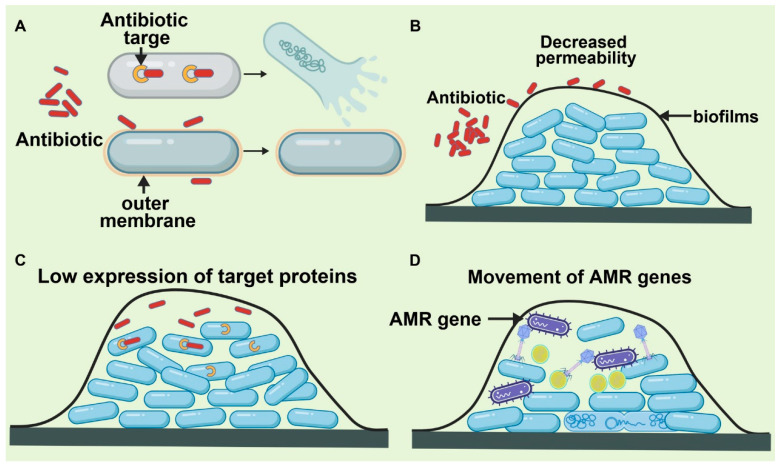
Resistance mechanisms mediated by the bacterial outer membrane and biofilm architecture. (**A**) The outer membrane functions as a selective permeability barrier that limits antibiotic penetration. (**B**) Biofilm-associated extracellular polymeric substances limit antimicrobial diffusion. (**C**) Downregulation or modification of intracellular targets reduces antibiotic efficacy. (**D**) Proximity in the biofilm facilitates horizontal transfer of AMR genes. The figure was created with BioRender.com.

Beyond intrinsic cellular defenses, bacteria often form surface-associated multicellular aggregates known as biofilms, which are a profoundly protective niche that severely limits antibiotic penetration [134] (Figure 5). Biofilm communities are encased in a self-produced extracellular polymeric substance (EPS), which is a matrix of exopolysaccharides, extracellular DNA (eDNA), proteins, and lipids that physically limits diffusion [135,136]. The development and maintenance of biofilms are frequently coordinated by quorum-sensing signaling systems, which regulate both biofilm architecture and virulence factor expression [137].

In the biofilm microenvironment, embedded bacteria enter a state of metabolic heterogeneity. A key subpopulation comprises per-sister cells, which are metabolically dormant variants that have exceptionally high tolerance to antibiotics that target active cellular processes. These cells enable survival of the population after treatment [138,139]. The dense aggregated architecture of biofilms promotes close cell-to-cell contact, which facilitates horizontal gene transfer (HGT). This process accelerates the dissemination of AMR genes in the community and compounds the overall resistance burden [138].

In summary, the synergistic interplay between the impermeable cellular envelope and the structured biofilm community results in a strong multi-layered defense strategy. The outer membrane functions as a selective molecular sieve, while the biofilm creates a diffusion-limited sanctuary that fosters tolerance and genetic exchange. This dual barrier undermines the efficacy of conventional antibiotics, and overcoming these pervasive resistance phenotypes requires a paradigm shift in therapy design. Future strategies should be multi-pronged and focus on: (1) enhancing drug penetration through outer membrane permeabilizers or siderophore-antibiotic conjugates; (2) disrupting biofilm integrity through matrix-degrading enzymes or quorum-sensing inhibitors; and (3) eradicating tolerant subpopulations by developing compounds that selectively target per-sister cell physiology. Only through such integrated approaches can we hope to effectively counter these pervasive barriers to antimicrobial therapy.

Biofilms and the outer membrane represent physical barriers. Phytochemicals are uniquely equipped to disrupt these barriers through surfactant-like properties (membrane permeabilization), inhibition of quorum sensing (preventing biofilm maturation), and degradation of extracellular polymeric substances. This multi-faceted attack on bacterial fortifications is a hallmark of plant derived anti-infective agents and is difficult for bacteria to counteract via single-point mutations.

## 3. Prospects for Phytochemicals and Their Derivatives to Combat AMR

The historical use of medicinal plants in treating infectious diseases is well documented and reflects their long-standing therapeutic value [140]. Plants produce a diverse arsenal of structurally complex secondary metabolites that often act on multiple microbial targets simultaneously. This polypharmacological profile enhances direct antibacterial efficacy and reduces the propensity for resistance evolution as pathogens would need to concurrently overcome several distinct mechanisms of action [141]. In the face of the escalating AMR crisis, this multi-target strategy contrasts with the single-target mode of many conventional antibiotics.

Medicinal plants are a rich and underexplored reservoir for the discovery of novel antibacterial agents and resistance-modifying adjuvants [142,143]. The search for plant-derived phytochemicals has grown and is being driven by the rising prevalence of MDR infections [144]. These compounds can interfere with bacterial viability through a wide range of mechanisms, including inhibition of EPs, disruption of nucleotide biosynthesis, compromise of cell membrane integrity, suppression of biofilm formation, and interference with key enzymatic systems [145,146,147]. By targeting several essential cellular processes concurrently, phytochemicals present a strong barrier to the evolution of bacterial resistance and are a promising sustainable means to reinvigorate the antimicrobial pipeline [148,149].

### 3.1. Inhibition or Disruption of Biofilm by Phytochemicals

#### 3.1.1. Biofilm as a Therapeutic Challenge

The development and pathogenicity of biofilms are often coordinated by quorum sensing (QS), which is a cell-density-dependent communication system that uses small signaling molecules to regulate gene expression, including genes related to virulence and EPS production [150]. The challenge is compounded in polymicrobial infections, where synergistic interactions can enhance the stability and maturation of the biofilm and amplify AMR [151]. Given the limitations of conventional antibiotics against biofilms, phytochemicals have emerged as promising anti-biofilm agents that can disrupt them through multiple complementary mechanisms.

#### 3.1.2. Targeting Biofilm Development and Integrity

Phytochemicals interfere with biofilm formation and stability through several key strategies:

(1) Quorum quenching: Phytochemicals function as potent quorum sensing inhibitors (QSIs) through precise molecular interventions [152,153]. For example, zingerone, a compound from ginger, structurally mimics native acyl-homoserine lactone (AHL) signals and competitively binds to the ligand-binding domain of the LasR receptor in *Pseudomonas aeruginosa*. This binding displaces the natural agonist and thereby blocks the transcription of virulence and biofilm genes. At sub-MIC concentrations (e.g., 100 µg/mL), zingerone reduces pyocyanin production and biofilm biomass by >50% without inhibiting bacterial growth, exemplifying a pure anti-virulence strategy [154]. Similarly, baicalin suppresses quorum sensing by downregulating the transcription of key QS regulators, including *lasI*, *lasR*, *rhlI*, *rhlR*, *pqsR*, and *pqsA*, effectively silencing both the Las and Rhl systems [155]. Caffeine also interferes with the LasR-LasI circuit in *P. aeruginosa*, reducing the secretion of elastase and other QS-dependent virulence factors [156]. These molecular mechanisms highlight how phytochemicals can selectively disrupt bacterial communication and virulence without exerting direct bactericidal pressure.
(2) Anti-adhesion strategies to prevent surface colonization: Surface attachment is a prerequisite for biofilm establishment, so blocking bacterial adhesion is a highly effective intervention [157]. Allicin and crude extracts of *Adiantum philippense* L. exhibit potent anti-adhesive activity that prevents initial surface colonization and reduces exopolysaccharide production [158,159]. Certain polyphenol extracts inhibit *Escherichia coli* adhesion, likely by interfering with fimbrial function (e.g., FimH) and perturbing cell surface hydrophobicity [160].
(3) Matrix degradation and disruption to undermine biofilm architecture: Beyond prevention, another important approach is degrading the mature EPS matrix, which is composed of polysaccharides, proteins, and eDNA. The benzophenanthridine alkaloid chelerythrine undermines biomass of *Staphylococcus aureus* biofilm by suppressing the synthesis of key matrix components (polysaccharides, proteins, eDNA) and preventing proper matrix assembly [161]. The sesquiterpene trans-trans-farnesol found in propolis inhibits early biofilm formation in *Streptococcus mutans* by increasing membrane proton permeability, suppressing glycolytic metabolism, and depleting the energy required for matrix production [162]. Notably, the action of such phytochemicals can be synergistically enhanced by combining them with enzymatic matrix-degrading agents (e.g., DNase), which could be a potent combinatorial strategy to dismantle established biofilms [163].

In summary, phytochemicals employ a multi-faceted anti-virulence strategy to combat biofilms by disrupting quorum-sensing networks, preventing bacterial adhesion, and degrading the structural matrix. This polypharmacological approach directly addresses the key vulnerabilities of biofilms: their coordinated development, structural integrity, and adaptive resilience. Thus, plant-derived compounds are highly promising candidates as standalone anti-biofilm agents and as potentiators of conventional antibiotics.

### 3.2. Bioactive Inhibitors Targeting Essential Bacterial Processes

#### 3.2.1. Alkaloids as Multifunctional Antimicrobial Agents

Alkaloids exert broad-spectrum antimicrobial activity through well-defined interactions with multiple essential bacterial targets, a feature that underpins their low resistance selection. The benzophenanthridine alkaloid sanguinarine, for example, intercalates into DNA and inhibits topoisomerase I, but also disrupts membrane potential and inhibits the MepA efflux pump in MRSA. This multi-target attack results in a MIC of 4–8 µg/mL against MRSA strains and, when combined with oxacillin, yields a synergistic FICI of 0.25–0.5, reducing the oxacillin MIC by 8 to 16 fold. Similarly, berberine inhibits DNA gyrase, interacts with FtsZ to disrupt cell division, and functions as an efflux pump substrate competitor, creating concerted antibacterial pressure. Their pharmacological efficacy arises from the chemical versatility and molecular flexibility of their heterocyclic frameworks, which enable simultaneous interactions with multiple bacterial targets [164]. For example, the benzophenanthridine alkaloid sanguinarine is isolated from plants such as *Argemone mexicana* L. and *Chelidonium majus* L. and demonstrates potent activity against MRSA by inhibiting bacterial replication and transcription [165]. Berberine is derived from *Berberis* species and has a multimodal mechanism that includes inhibition of DNA gyrase/topoisomerase, interference with RNA polymerase function, and disruption of cell division machinery [166].

The isoquinoline alkaloid ungeremine from *Pancratium illyricum* L. primarily exhibits antibacterial activity by inhibiting topoisomerase and inducing DNA cleavage [167]. Quinoline alkaloids, such as dictamnine, kokusagine, and maculine from *Teclea afzelii*, specifically target type II topoisomerase and its associated enzymes and effectively blocks DNA replication [168]. Tomatidine, a steroidal alkaloid from solanaceous plants, shows antibacterial activity against *Listeria*, *Bacillus*, and *Staphylococcus* species, and emerging evidence points to ATP synthase inhibition as its primary mechanism [169,170].

#### 3.2.2. Polypharmacological Inhibition of Bacterial Enzymes

Beyond alkaloids, diverse plant-derived secondary metabolites target essential bacterial enzymes through polypharmacological mechanisms. The catechin derivative epigallocatechin gallate (EGCG) exemplifies this approach by competitively occupying the ATP-binding site of DNA gyrase (GyrB subunit), suppressing efflux-pump activity, and downregulating chromosomal penicillinase expression [171]. Chebulinic acid from *Terminalia chebula* Retz. maintains efficacy against quinolone-resistant *Mycobacterium tuberculosis* due to a unique DNA gyrase binding mode that bypasses common resistance mutations [172]. Allicin is a broad-spectrum inhibitor of sulfhydryl-dependent enzymes, including alcohol dehydrogenase, thioredoxin reductase, and RNA polymerase, which leads to comprehensive disruption of bacterial redox homeostasis and transcriptional regulation [173]. Taxifolin is a phenolic compound from *Allium cepa* L. that potently inhibits beta-ketoacyl-acyl carrier protein synthase (FabF), a key enzyme in bacterial fatty-acid biosynthesis, and demonstrates potent activity against *Enterococcus faecalis* [174].

#### 3.2.3. Overcoming Target-Based Resistance Mechanisms

A critical challenge in antimicrobial therapy involves bacterial modification of antibiotic target sites, which reduces drug binding affinity. Plant-derived compounds provide innovative strategies to circumvent this resistance by modulating the expression or function of modified targets. Proanthocyanidins from *Vaccinium macrocarpon* Aiton synergize with levofloxacin against *Hellicobacter pylori*, partially through downregulation of the synthesis of penicillin-binding protein 2a (PBP2a), a key determinant of β-lactam resistance [175]. Similarly, phenolic extracts from *Verbena officinalis* L., *Magnolia officinalis*, *Daphne genkwa*, and *Momordica charantia* resensitize MRSA to oxacillin, which primarily involves transcriptional inhibition of PBP2a and PBP4 expression [176]. Structural and proteomic investigations have shown that these phytochemicals frequently interact with allosteric or regulatory sites of target proteins, which induces conformational changes that either restore antibiotic binding capacity or impair target protein function [177]. This sophisticated mode of action highlights the potential of plant-derived compounds as next-generation adjuvants that could restore susceptibility to conventional antibiotics against MDR pathogens.

#### 3.2.4. Structural Insights and Mechanistic Implications

Recent advances in structural biology and computational modeling have yielded new insights into the molecular interactions between phytochemicals and bacterial targets. High-resolution crystallographic studies show that many bioactive plant compounds engage conserved structural motifs in essential enzymes through intricate networks of hydrogen bonding, π–π stacking, and hydrophobic interactions. These mechanisms often diverge substantially from those of conventional antibiotics [17,178]. For instance, molecular docking analyses demonstrate that specific flavonoid derivatives establish multipoint contacts in the substrate-binding pockets of both DNA gyrase and topoisomerase IV, which is responsible for their capacity for dual-target inhibition [179]. Kinetic studies using surface plasmon resonance (SPR) and isothermal titration calorimetry (ITC) have quantified these interactions. The results show that certain alkaloids bind to ribosomal subunits with dissociation constants in the low micromolar range, which correlates well with their observed antimicrobial potency [180,181,182].

These mechanistic insights validate traditional ethnopharmacological applications and provide rational frameworks for structure-based optimization of lead compounds. Particularly noteworthy is the emerging recognition that many phytochemicals exhibit “moonlighting” capabilities of binding to multiple structurally distinct targets, which may underlie their lower propensity for resistance development compared to single-target antibiotics. This poly-pharmacological profile and frequent engagement of allosteric regulatory sites make plant-derived inhibitors valuable templates for developing novel antimicrobial agents that operate through unconventional mechanisms that could potentially bypass resistance pathways.

### 3.3. Medicinal Plant Compounds as EP Inhibitors

#### 3.3.1. EPs as a Key Target for Phytochemicals

The overexpression of bacterial EPs is a primary determinant of pathogens’ ability to actively expel a wide spectrum of antibiotics and decrease intracellular drug concentrations [177,183]. Medicinal plants are a chemically diverse and largely unexplored repository of bioactive compounds that can target these systems. Plant-derived EP inhibitors (phyto-EPIs) are promising for adjunctive strategies to potentiate conventional antibiotics, resensitize resistant bacterial strains, and restore clinical efficacy against MDR infections. The strategic inhibition of efflux machinery directly counteracts a key resistance mechanism, reduces the effective doses of co-administered antibiotics, and could potentially mitigate toxicity and slow further resistance development.

#### 3.3.2. Alkaloids as Potent EP Inhibitors

Alkaloids are one of the most pharmacologically significant classes of plant-derived EPIs and have potent activity against diverse EP families [184,185]. The indole alkaloid reserpine serves as a prototypical efflux pump inhibitor (EPI), primarily targeting MFS transporters. It acts as a competitive inhibitor by binding directly to the substrate-binding site of the Tet(K) pump in *Bacillus subtilis* and the NorA pump in *Staphylococcus aureus*. This binding enhances intracellular tetracycline accumulation by 4 fold in resistant strains, thereby restoring antibiotic susceptibility. Its mechanism differs from that of synthetic efflux pump inhibitors, as it does not require metabolic activation and binds with high affinity to a conserved site within the pump’s transmembrane domain. Mechanistic studies on *Bacillus subtilis* demonstrate that reserpine enhances tetracycline efficacy by directly binding to critical residues in the Tet efflux transporter and physically obstructing the antibiotic extrusion channel [186]. The piperidine alkaloid piperine from *Piper nigrum* L. acts synergistically with fluoroquinolones against MRSA, which primarily occurs through inhibition of MFS-type EPs, and this activity is conserved in its synthetic structural analogs [187].

Other alkaloids such as specific indole derivatives synergize with tetracycline by transcriptionally downregulating EP gene expression in clinical isolates of *Escherichia coli* [188]. Steroidal alkaloids such as tomatidine and conessine function as broad-spectrum potentiators that enhance the activity of diverse antibiotics (including ampicillin and ciprofloxacin) against pathogens including *Pseudomonas aeruginosa* and *Enterococcus faecalis* [189,190]. Notably, conessine shows specific inhibitory activity against RND-family pumps in *Acinetobacter baumannii* [191,192], which highlights the structural specificity in this compound class.

#### 3.3.3. Phenolics and Flavonoids for Multifunctional Efflux Modulation

Phenolic compounds and flavonoids are another major class of effective phyto-EPIs and often exhibit multifunctional mechanisms that extend beyond simple pump blockade. The stilbenoid resveratrol inhibits multidrug efflux across phylogenetically diverse species and affects ABC transporters in *Campylobacter jejuni* and *Mycobacterium smegmatis* [192]. In *A. baumannii*, resveratrol restores susceptibility to chlorhexidine by modulating RND-pump expression [193], while in *S. aureus*, it reverses norfloxacin resistance by suppressing MFS-type efflux activity [194].

The flavonoid baicalein is a potent inhibitor of the MFS efflux pump NorA. Mechanistic studies indicate that it binds competitively to the hydrophobic drug binding pocket of NorA, directly blocking the extrusion of fluoroquinolones. At a non-bactericidal concentration of 20 µg/mL, baicalein reduces the ciprofloxacin MIC against a NorA overexpressing MRSA strain from 32 µg/mL to 4 µg/mL, yielding a fractional inhibitory concentration index (FICI) of 0.28. Additionally, baicalein’s planar molecular structure enables it to disrupt membrane fluidity, resulting in a dual action potentiation effect that is difficult for bacteria to circumvent via single point mutations in the efflux pump gene. [195,196]. The isoflavonoid biochanin A suppresses efflux in MRSA through transcriptional downregulation of the NorA efflux-pump protein [197]. Similarly, kaempferol and its glycoside derivatives enhance ciprofloxacin activity against *S. aureus* through MFS-pump inhibition [198], and the anti-MRSA activity of EGCG is partly due to this efflux-inhibitory mechanism [171]. The structural diversity in this class encompassing simple phenolics and complex flavonoids enables interactions with various EP components through hydrogen bonding, π-π stacking, and hydrophobic interactions.

#### 3.3.4. Diverse Phytochemicals with Efflux-Inhibitory Activity

Numerous other plant-derived compounds demonstrate notable efflux-inhibitory properties through distinct molecular mechanisms. The ergot alkaloid lysergol and its synthetic derivatives interfere with ATP-dependent efflux, as evidenced by direct ATPase inhibition and transcriptional downregulation of genes encoding ABC transporter subunits [199]. Nitrile glycosides from *Moringa oleifera* pods downregulate key efflux-pump genes (*acrB*, *yojI*), which occurs alone or in synergy with tetracycline [200]. The diterpene alcohol phytol and related compounds inhibit ABC transporters by suppressing the expression of *yojI*, which encodes a multidrug efflux ATP-binding protein [201,202]. These examples underscore the remarkable chemical diversity of plant-derived EPIs and their capacity to engage efflux machinery through multiple inhibitory strategies, including direct pump blockade, interference with energy coupling, and transcriptional regulation of pump expression.

Bacterial EPs are a primary defensive mechanism that confer broad-spectrum antibiotic resistance and enhance microbial survival under therapeutic stress [203]. Given that most antibiotic classes remain vulnerable to efflux-mediated resistance, the development of potent specific EPIs is a crucial strategy for revitalizing the antimicrobial armamentarium. Medicinal plants provide a rich, sustainable, and chemically diverse source of such compounds called phyto-EPIs.

The preceding analysis reveals that phytochemicals counteract antimicrobial resistance (AMR) not through a single dominant mechanism, but rather through inherent polypharmacology. A single compound such as curcumin, for instance, can act simultaneously to: (i) inhibit β-lactamase activity via competitive binding or metal-chelation; (ii) disrupt membrane integrity through hydrophobic interactions; and (iii) suppress quorum sensing by downregulating the lasI/R system. This multi-faceted attack establishes a high genetic barrier to the development of resistance. In contrast, conventional antibiotics often fail precisely because of their exquisite specificity: a single point mutation in the drug target (e.g., gyrA for fluoroquinolones) or acquisition of a single hydrolytic enzyme (e.g., a β-lactamase) can be sufficient to confer resistance. Phytochemicals, by engaging multiple targets with moderate affinity, impose a distributed physiological stress that is evolutionarily more costly for bacteria to overcome. This fundamental distinction in strategic approach network perturbation versus target specific inhibition underpins the value of phytochemicals as resistance modulating agents and as templates for the design of next generation antimicrobials. Research should prioritize the systematic identification of novel phyto-EPI leads and structural optimization through medicinal chemistry approaches to enhance potency, selectivity, and pharmacokinetic properties. Detailed mechanistic elucidation using structural biology, molecular modeling, and omics technologies will be essential for understanding their precise modes of action and potential for synergy with conventional antibiotics. The development of these plant-derived compounds as adjunctive therapies alongside standard antimicrobial regimens is a viable and sustainable strategy to counteract the escalating global threat of MDR infections. Such efforts could potentially extend the clinical lifespan of existing antibiotics while informing the design of next-generation antimicrobial agents.

## 4. Future Directions and Translational Potential of Medicinal Plants

### 4.1. A Paradigm Shift in Antimicrobial Strategy

Medicinal plants are increasingly recognized as a promising source of next-generation resistance-modifying agents. Research has strategically evolved beyond the singular pursuit of novel bactericidal phytochemicals toward harnessing these compounds as antibiotic adjuvants and pathogenesis disruptors. This paradigm shift is leveraging the intrinsic polypharmacology of plant-derived molecules and their ability to simultaneously engage multiple bacterial targets. This creates a higher genetic barrier to resistance development compared to conventional single-target antibiotics.

### 4.2. Emerging Strategies

#### 4.2.1. Resistance Reversal Through Antibiotic Potentiation

Current research is converging on four distinct yet complementary strategic approaches to utilize medicinal plants in combating AMR. Rather than focusing solely on novel antibiotic discovery, a primary strategy is to revitalize existing antibiotics for which efficacy has been compromised by resistance. This is achieved by co-administering phytochemicals that block specific resistance mechanisms.

For instance, alkaloids such as reserpine and flavonoids like baicalein act as potent EP inhibitors that increase intracellular antibiotic accumulation. Compounds such as aspergillomarasmine A (originally from *Aspergillus*) and certain plant extracts exhibit β-lactamase inhibitory activity [204]. Administering these “phyto-adjuvants” alongside conventional antibiotics can significantly reduce the minimum inhibitory concentration (MIC) against MDR pathogens, including MRSA and carbapenem-resistant *Acinetobacter baumannii*. They could thus restore clinical efficacy and extend the therapeutic lifespan of existing drugs.

#### 4.2.2. Anti-Virulence and Anti-Biofilm Therapeutics

The “disarmament” strategy involves attenuating bacterial pathogenicity and resilience without directly challenging microbial survival, which minimizes the selection pressure that drives resistance. Phytochemicals such as hamamelitannin (from witch hazel) and specific quinones disrupt QS communication, which effectively attenuates the production of toxins, proteases, and other virulence factors [205]. Others such as ursolic acid and various plant lectins potently inhibit biofilm formation, which is a critical factor in chronic infections and antibiotic tolerance [206]. By reducing virulence and preventing community-structured growth, this approach enhances host immune clearance and antibiotic accessibility.

#### 4.2.3. Inhibition of Horizontal Gene Transfer (HGT)

Another approach is to target the dissemination of resistance genes. Compounds like plumbagin and the naphthoquinone acetylshikonin have demonstrated the ability to inhibit the conjugative transfer of resistance plasmids (e.g., those harboring *mcr-1* or *blaNDM-1* genes) between bacterial cells [207,208]. By blocking this critical route of HGT, such inhibitors could contain the environmental and gut microbiota spread of resistance determinants and address the AMR crisis at the epidemiological transmission level.

#### 4.2.4. Plasmid Curing for Genetic Re-Sensitization

A radical genetics-based strategy involves the selective elimination of resistance-encoding plasmids from bacterial populations in a process known as “curing.” Certain plant secondary metabolites, including specific berberine derivatives and extracts from *Achyranthes aspera*, can promote the loss of these plasmids [209,210]. This process reverts phenotypically resistant bacterial strains to a susceptible state, effectively erases a key genetic determinant of resistance, and restores susceptibility to first-line antibiotics. This approach is a powerful means of reversing established resistance in a bacterial population.

### 4.3. Translational Challenges and Innovative Solutions

Despite the significant potential, the translational development of plant-based antimicrobials faces considerable hurdles. These include the inherent chemical complexity of plant extracts, which hampers standardization, as well as suboptimal pharmacokinetic profiles (e.g., low oral bioavailability, rapid metabolism and elimination), and potential host cytotoxicity at therapeutically effective concentrations. Future progress depends on the integration of multidisciplinary technologies:

1. Synthetic biology and metabolic engineering to optimize and sustainably mass-produce promising phytochemical scaffolds.
2. Medicinal chemistry for rational structural modification to enhance potency, selectivity, and drug-like properties of lead compounds.
3. Advanced drug-delivery systems using nanotechnologies such as liposomal encapsulation, polymeric nanoparticles, and conjugate systems to overcome limitations in bioavailability, stability, and targeted delivery.

### 4.4. Precision Phytotherapy

By systematically addressing the chemical, biological, and pharmacological barriers, medicinal plants can be translated into the next generation of precision tools for AMR management. The future lies not in merely rediscovering plant antibiotics, but also in repurposing and optimizing phytochemicals as resistance-breaking adjuvants, virulence attenuators, and gene transfer blockers. This approach could position plant-derived compounds as indispensable components of a sustainable and innovative arsenal in the ongoing battle against MDR infections.

## 5. Conclusions

AMR is a critical and growing threat to global public health that is driven by the sophisticated and multifactorial resistance mechanisms developed by bacterial pathogens. This review has systematically examined the key molecular pathways underlying bacterial antibiotic resistance. These include the enzymatic inactivation of drugs (e.g., through β-lactamase-mediated hydrolysis), modification of antibiotic targets (e.g., through ribosomal mutations or altered penicillin-binding proteins), reduced membrane permeability, and the overexpression and activity of multidrug EPs. These diverse mechanisms challenge the conventional “one drug, one target” paradigm and underscore the urgent need for innovative adjunctive therapeutic strategies.

In this context, botanical sources and their derived phytochemicals are a promising frontier for alternative antimicrobials and resistance-modifying agents. As inhibitors of biofilm formation and stability, they neutralize a key virulence factor that shields bacterial communities and limits antibiotic penetration in chronic infections. As direct inhibitors of resistance-conferring enzymes (e.g., β-lactamases, DNA topoisomerases), they could potentially restore the efficacy of compromised antibiotics, and most notably, they are potent EPIs. Specific plant-derived alkaloids, flavonoids, and terpenoids can effectively block EP function, increase intracellular antibiotic accumulation, and resensitize MDR strains to conventional drugs. This EPI strategy could be a direct route to rejuvenating our antibiotic arsenal.

Translating phytochemical potential into clinical tools requires a focused interdisciplinary approach. Strategic priorities must include bioactivity-guided discovery, structural characterization, and medicinal chemistry optimization of lead compounds to enhance their potency, selectivity, and pharmacokinetic properties. Comprehensive mechanistic studies should also employ integrated omics (genomics, proteomics, metabolomics) and computational modeling to elucidate the polypharmacology and synergy with standard antibiotics. Advanced delivery systems also need to be developed, including engineered nanoparticles or lipid-based carriers to overcome limitations in solubility, stability, targeted delivery, and bioavailability while mitigating potential off-target effects. Ultimately, the integration of phytochemical-based adjuncts with conventional antibiotics is a viable and sustainable strategy to outmaneuver bacterial adaptation, prolong the utility of existing drugs, and bolster long-term defenses against MDR infections.

## Data Availability

No new data were created or analyzed in this study. Data sharing is not applicable to this article.
